# Vitamin D Receptor rs731236 Polymorphism Modulates Cancer Cachexia Susceptibility and Overall Survival: A Real-World Study on Context-Dependent Vitamin D Signalling

**DOI:** 10.3390/ijms27135816

**Published:** 2026-06-27

**Authors:** Valéria Tavares, Ana Carolina Leão Silva, Anna Flávia Xavier, Inês Guerra de Melo, Tiago Ferreira, Mariana Moreira Pires, Cláudia Silva, Virgínia Rocha Dias, Maria Paula Silva, Joana M. O. Santos, Rui Medeiros

**Affiliations:** 1 Molecular Oncology and Viral Pathology Group, IPO Porto Research Centre (CI-IPOP), Portuguese Oncology Institute of Porto (IPO Porto)/Department of Pathology and Laboratory Medicine/RISE-Associate Laboratory (Health Research Network)/Porto Comprehensive Cancer Centre Raquel Seruca (Porto.CCC), 4200-072 Porto, Portugal; valeria.tavares@ipoporto.min-saude.pt (V.T.); acleao.nutri@gmail.com (A.C.L.S.); annaflaxm@gmail.com (A.F.X.); ines.melo@ipoporto.min-saude.pt (I.G.d.M.); tiagoterras55@gmail.com (T.F.); marianamp006@gmail.com (M.M.P.); joana.oliveira.santos@ipoporto.min-saude.pt (J.M.O.S.); 2 Research Department, Portuguese League Against Cancer (NRNorte), 4200-172 Porto, Portugal; 3 School of Medicine and Biomedical Sciences (EMCB), Fernando Pessoa University, 4420-096 Gondomar, Portugal; 4 Faculty of Medicine, University of Porto (FMUP), 4200-072 Porto, Portugal; 5 Centre for the Research and Technology of Agro-Environmental and Biological Sciences (CITAB), University of Trás-os-Montes and Alto Douro (UTAD), 5000-801 Vila Real, Portugal; 6 Day Hospital, Portuguese Oncology Institute of Porto (IPO Porto), 4200-072 Porto, Portugal; claudia_ssilva@hotmail.com (C.S.); virgi.dias@hotmail.com (V.R.D.); 7 Palliative Care Service, Portuguese Oncology Institute of Porto (IPO Porto), 4200-072 Porto, Portugal; mpaulasilvajc@gmail.com; 8 Faculty of Health Sciences, Fernando Pessoa University, 4200-150 Porto, Portugal; 9 Instituto de Ciências Biomédicas Abel Salazar (ICBAS), University of Porto, 4050-313 Porto, Portugal; 10 European Cancer Organisation (ECO), 1040 Brussels, Belgium

**Keywords:** VDR, cachexia, neoplasms, polymorphism, single nucleotide, biomarkers

## Abstract

Cancer-associated cachexia (CAC) is a complex metabolic syndrome characterised by progressive skeletal muscle loss, systemic inflammation, reduced treatment tolerance, and poor survival. Marked interindividual variability in CAC susceptibility suggests that host genetic factors may contribute to its development. Vitamin D plays an important role in muscle metabolism and inflammatory control through activation of the vitamin D receptor (VDR). VDR signalling influences myogenesis, mitochondrial function, insulin-like growth factor pathways, and pro-inflammatory cytokine expression, all of which are implicated in CAC pathogenesis. Hence, *VDR* variants, including the rs731236 (A>G) polymorphism, which modifies receptor activity, may affect CAC pathogenesis. Thus, this study investigated the association between the polymorphism and susceptibility to CAC as well as patient survival in a cohort of 140 adult cancer patients. Briefly, the rs731236 GG genotype was significantly associated with a lower prevalence of CAC across disease diagnostic approaches (chi-square tests, *p* < 0.05). Furthermore, GG genotype carriers demonstrated significantly improved survival compared with carriers of AA/AG genotypes (Log-rank and Tarone–Ware tests, *p* < 0.05). In summary, these findings suggest that the rs731236 polymorphism influences both susceptibility to CAC and survival outcomes in patients with cancer, further supporting a clinically relevant role for vitamin D signalling in supportive oncology care.

## 1. Introduction

Cancer-associated cachexia (CAC) is a multifactorial metabolic syndrome characterised by involuntary weight loss, progressive skeletal muscle wasting, systemic inflammation, and functional impairment [1]. It affects up to 80% of patients with advanced malignancies, including both solid and haematological tumours, and is a major contributor to treatment intolerance, diminished quality of life, and increased mortality [2,3]. Unlike simple starvation, CAC is driven by complex metabolic and inflammatory alterations that are not fully reversible by nutritional support alone [4]. Despite its high prevalence and prognostic relevance, with 20% of cancer patients dying due to this condition, CAC remains underdiagnosed, undertreated, and insufficiently stratified in routine oncology practice [2,3].

Chronic inflammation is a central driver of CAC pathophysiology, promoting muscle proteolysis, impairing protein synthesis, causing mitochondrial dysfunction, and altering overall energy metabolism. Pro-inflammatory cytokines such as interleukin-6 (IL-6) and tumour necrosis factor-α (TNF-α) activate catabolic signalling pathways, disrupt anabolic responses, and contribute to endocrine resistance, ultimately accelerating muscle loss [1,4]. However, substantial interindividual variability exists in the development and severity of CAC, suggesting that several host-related factors, including genetic determinants, may modulate susceptibility to this syndrome [5].

Vitamin D3 (or simply vitamin D) plays a fundamental role at the interface between endocrine and immune regulation, exerting biological effects that extend well beyond its traditional involvement in calcium and phosphate metabolism [6]. Namely, it has emerged as a central modulator of muscle function, metabolism and immune regulation, primarily via activation of the vitamin D receptor (VDR), a nuclear transcription factor expressed in skeletal muscle, immune cells, and adipose tissue. VDR signalling regulates myogenesis, muscle fibre composition, mitochondrial oxidative capacity, insulin-like growth factor 1 (IGF-1) pathways, and the production of inflammatory cytokines [7,8,9,10]. Consistently, vitamin D deficiency and impaired VDR signalling have been associated with sarcopenia (progressive loss of skeletal muscle mass associated with ageing), frailty, and chronic inflammatory states, all of which share pathophysiological features with CAC [11,12,13].

Worldwide, genetic polymorphisms are the most prevalent genetic variations, with implications for several clinical traits [14,15]. Genetic polymorphisms in *VDR* may influence the receptor’s transcriptional activity and messenger (mRNA) stability, thereby modifying downstream biological responses to vitamin D. The rs731236 (A>G) variant, also known as TaqI, is one of the most studied *VDR* polymorphisms due to its influence on vitamin D signalling [16,17]. It is a single-nucleotide polymorphism (SNP) located in the 3′ untranslated region (UTR) of exon 9 of the *VDR* gene on chromosome 12q13.11 [18,19]. It involves the substitution of adenine with guanine (or, equivalently, thymine with cytosine, depending on strand orientation) [19]. Although *VDR* rs731236 does not alter the amino acid sequence of the encoded protein (i.e., it is a synonymous variant), it may nonetheless affect gene regulation and expression through mechanisms such as changes in transcription, splicing, co-translational folding, and mRNA stability, among other functionally relevant alterations [20]. Given its link with altered inflammatory profiles, muscle-related phenotypes, and clinical outcomes, rs731236 has been widely studied across multiple conditions and traits, including musculoskeletal diseases [21,22,23], neurological disorders [24,25], infectious and immune-related diseases [26,27,28,29], gut microbiota composition [30], cardiometabolic conditions [31,32] and reproductive and endocrine disorders [33,34,35]. Furthermore, the influence of *VDR* rs731236 has been evaluated across multiple malignancies with respect to disease susceptibility and prognosis [36,37,38,39,40,41]. However, the associations are frequently inconsistent across studies, ethnic groups and disease contexts, requiring additional studies for validation. Regarding CAC, the role of *VDR* rs731236 remains poorly explored, with most previous studies focusing on either vitamin D levels or single tumour types, rather than the intersection of host genetics, muscle wasting, and patient survival. Given existing knowledge gaps, this study explored the association of this polymorphism with susceptibility to CAC and survival outcomes in a cohort of 140 adult cancer patients from the Northern region of Portugal.

## 2. Results

### 2.1. Distribution of VDR rs731236 Genotypes

**Figure 1:**
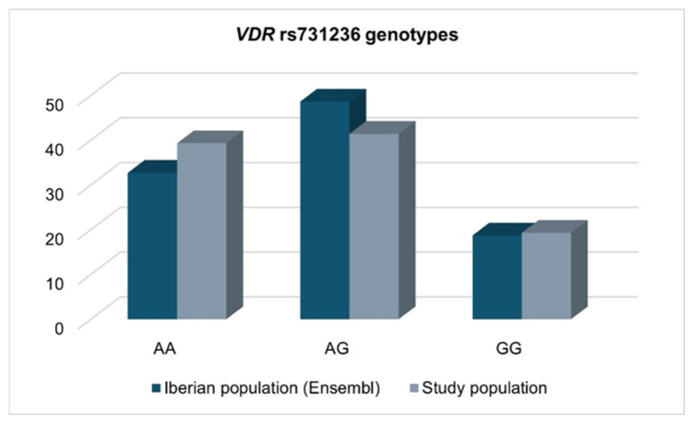
Genotype distribution in the study cohort compared with the reference Iberian population (https://www.ensembl.org/index.html, last accessed on the 28 December 2025).

Genotype distributions for *VDR* rs731236 in the study population and the reference Iberian population described in the Ensembl database (https://www.ensembl.org/index.html, last accessed on the 28 December 2025) are indicated in Figure 1. Compared with the reference population, the distribution of SNP genotypes did not differ significantly [Chi-square (χ^2^) test, *p* = 0.551], indicating that the variant is consistent with Hardy–Weinberg equilibrium (HWE) expectations. Notably, the minor allele frequency (MAF) was 43% and 40% in the reference population and in the study population, respectively.

Notably, SNP genotype distributions did not differ significantly depending on patient demographic or clinicopathological variables (χ^2^ test or Fisher’s exact test, *p* > 0.05).

### 2.2. Association Between VDR rs731236 and CAC Status

**Figure 2:**
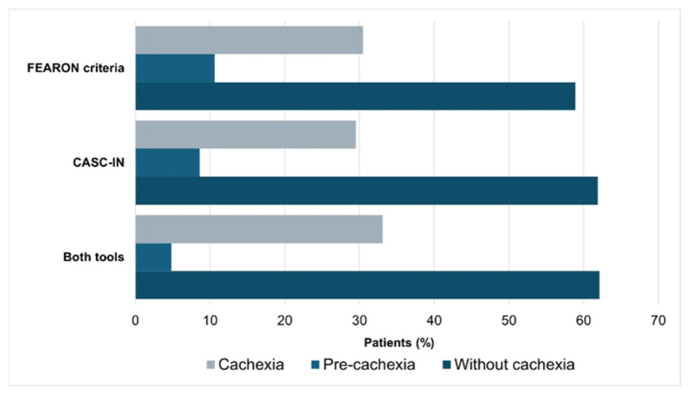
Cachexia prevalence according to each diagnostic approach.

Cachexia prevalence in the study cohort was approximately 30% using Fearon criteria, CASC-IN, or the combination of both tools in disease diagnosis (Figure 2).

Associations between the *VDR* rs731236 SNP and CAC susceptibility were examined under codominant, dominant and recessive genetic models. Analyses were conducted using the three diagnostic approaches (Fearon criteria, CASC-IN tool and the combination of both tools), considering (i) three-category comparisons (no CAC, pre-CAC, and CAC) and (ii) dichotomised comparisons (no CAC versus pre-CAC + CAC). A significant association was observed between *VDR* rs731236 genotypes and CAC status (χ^2^ test or Fisher’s exact test, *p* < 0.05). Namely, across all diagnostic criteria, the SNP GG genotype was consistently more prevalent among individuals without CAC (Table 1).

Consistently, when pre-CAC and CAC patients were combined into a single entity group, the significant association between *VDR* rs731236 genotypes and CAC status remained (Table 2). Under the recessive genetic model (GG vs. AG/AA), for which significant results were observed, the GG genotype was associated with a significantly lower disease risk according to the Fearon criteria [odds ratio (OR) = 0.27; 95% confidence interval (CI), 0.09–0.75], the CASC-IN criteria (OR = 0.32; 95% CI, 0.11–0.90), and the combined assessment tool (OR = 0.20; 95% CI, 0.06–0.71).

### 2.3. Association Between VDR rs731236 and Patient Survival

**Figure 3:**
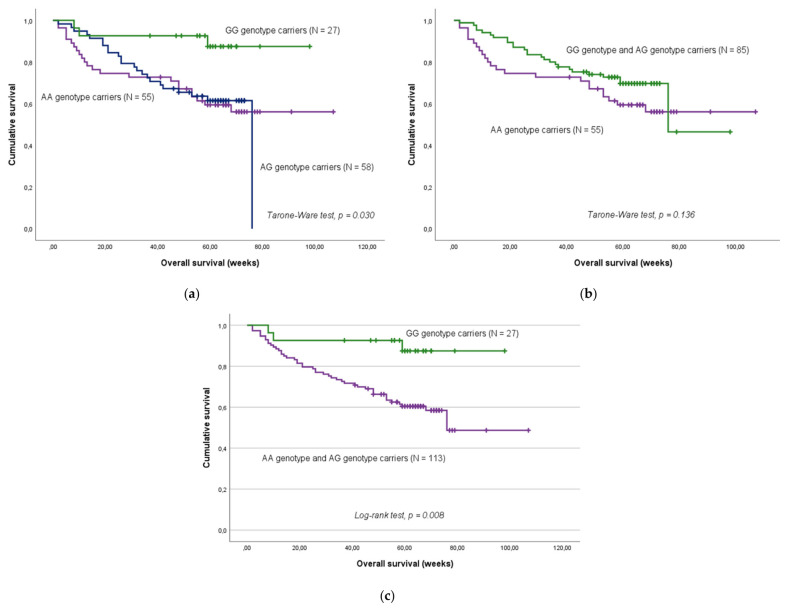
Overall survival (OS) according to *VDR* rs731236 genotypes. (**a**) In the codominant genetic model, patients with the GG genotype had significantly higher survival time compared to AA and AG genotype carriers (mean OS: 89.4 ± 4.8 weeks, 71.9 ± 5.8 weeks, and 57.6 ± 3.4 weeks, respectively; Tarone–Ware test, *p* = 0.030). (**b**) In the dominant genetic model, no significant differences in survival time were observed (Tarone–Ware test, *p* = 0.136). (**c**) In the recessive genetic model, patients carrying the GG genotype had significantly longer survival time than A allele carriers (mean OS = 89.4 ± 4.8 weeks and 71.2 ± 4.6 weeks, respectively; Log-rank test, *p* = 0.008).

In the overall cohort (N = 140), carriers of the SNP GG genotype exhibited a significantly longer overall survival (OS) than patients with AA/AG genotypes (Figure 3). Individuals harbouring the GG genotype experienced a 76% reduction in the risk of death compared with their counterparts [hazard ratio (HR) = 0.24; 95% CI, 0.07–0.76; *p* = 0.016]. This association remained robust after adjustment for potential confounding variables, confirming the independent prognostic value of the *VDR* rs731236 GG genotype (Table 3). Notably, these analyses were based on 49 events (deaths).

To specifically assess the impact of *VDR* rs731236 on survival in CAC patients, a subgroup analysis was performed. In this CAC-only population, no significant association between SNP genotypes and survival was detected, irrespective of the diagnostic criteria or genetic model applied (Log-rank and Tarone–Ware tests, *p* > 0.05).

## 3. Discussion

Muscle regeneration is a multifaceted process that requires the recovery of mitochondrial activity and the activation of satellite cells, which are the intrinsic stem cells responsible for skeletal muscle repair. In recent years, growing evidence has implicated vitamin D signalling as an important regulator of skeletal muscle metabolism, regeneration, and protein turnover, pathways that are critically disrupted in CAC and that influence patient outcomes [10]. In this context, the present study investigated the association between the *VDR* rs731236 polymorphism and clinical outcomes in cancer patients, focusing on CAC susceptibility and OS. Our findings indicate that GG genotype carriers have a lower risk of developing cachexia and demonstrate improved OS, independent of CAC status, compared with individuals carrying the AA or AG genotypes. To understand how the GG genotype may confer protection against CAC, it is necessary to consider the role of vitamin D in skeletal muscle.

Vitamin D is a pleiotropic hormone primarily produced in the skin through ultraviolet-dependent synthesis. It is subsequently converted in the liver into 25-hydroxyvitamin D, the primary circulating form, and then further hydroxylated to its active form, 1,25(OH)_2_D_3_. Although this final activation step primarily occurs in the kidneys, several other tissues also possess the enzymatic machinery required for local vitamin D activation [11]. In the circulation, 1,25(OH)_2_D_3_ is transported to target tissues by vitamin D-binding proteins. Its biological effects are mainly mediated by its receptor, VDR, a widely expressed nuclear receptor [42]. Upon ligand binding, the VDR heterodimerises with the retinoid X receptor and regulates gene transcription via interactions with vitamin D response elements (VDREs) within promoters of target genes involved in calcium and bone metabolism, cell growth and differentiation, and immune response and function [43,44]. In addition to these classic genomic actions, vitamin D also exerts rapid non-genomic effects via key signalling cascades, including the mitogen-activated protein kinase (MAPK) cascade, the phosphoinositide 3-kinase/protein kinase B (PI3K-Akt) pathway, and the mechanistic target of rapamycin (mTOR) signalling axis, which intersect with metabolic and anabolic networks governing muscle mass and function, namely modulation of mitochondrial function and intracellular calcium handling [10,45,46].

Within skeletal muscle, VDR expression is dynamic and stage-specific, being most abundant in proliferating myoblasts and regenerating fibres and minimal in mature myofibres. This distribution suggests that vitamin D primarily regulates muscle regeneration and satellite cell activity rather than the contractile function of fully differentiated muscle [47,48,49,50]. Consistently, 1,25(OH)_2_D_3_ modulates myogenesis by limiting early myoblast proliferation through cell-cycle arrest, while promoting later differentiation and myotube formation under specific conditions. At the molecular level, vitamin D influences the expression of key regulators of muscle mass, including Forkhead box O (FOXO) transcription factors, IGF-1 signalling components, myostatin, and integrins, thereby coordinating protein synthesis, degradation and repair processes [46,51,52]. The transcriptional activity of these pathways is further modulated by multiple coactivators and corepressors, enabling tissue- and context-specific regulation of gene expression [11,42].

Under physiological conditions, vitamin D is well recognised for its anti-inflammatory and anabolic effects, including suppression of pro-inflammatory cytokines and inhibition of the ubiquitin-proteasome pathway, which drives muscle protein breakdown, while promoting muscle regeneration via protein synthesis [10,53,54]. Salles et al. (2013) demonstrated that vitamin D enhances muscle cell responsiveness to insulin and leucine [53]. Similarly, Garcia et al. (2011) showed that vitamin D promotes muscle cell maturation, supporting muscle regeneration [54]. These studies indicate that vitamin D supports muscle homeostasis, and its deficiency may predispose to muscle atrophy.

Intriguingly, the relationship between VDR signalling and muscle homeostasis appears to be more complex in the setting of CAC, which is characterised by chronic systemic inflammation, metabolic dysregulation, and impaired regenerative capacity [1,4]. Pro-inflammatory cytokines elevated in CAC, including TNF-α and IL-6, directly promote proteolysis while suppressing anabolic signalling, thereby accelerating muscle loss. Within this inflammatory environment, dysregulation of VDR may paradoxically exacerbate muscle wasting rather than preserve muscle mass. Camperi et al. (2017) provided critical evidence of this molecular paradox using tumour-bearing animal models and human muscle biopsies [11]. They found that while circulating vitamin D levels decrease significantly in CAC, *VDR* expression in skeletal muscle simultaneously increases. Although this rise was initially interpreted as a compensatory response to low serum vitamin D levels, supplementation failed to reverse the muscle loss and instead further elevated *VDR* expression, suggesting a maladaptive feedback loop. The authors proposed that under the inflammatory conditions of CAC, vitamin D signalling is reprogrammed to act as a molecular brake on myogenic differentiation by suppressing myogenin, a key regulator of muscle fibre formation, effectively stalling regeneration. Consequently, vitamin D supplementation in this context may be counterproductive, as it can exacerbate *VDR* overexpression without restoring muscle repair [11,55]. Collectively, these findings suggest that the biological effects of vitamin D are highly context-dependent and that dysregulated VDR activity, rather than vitamin D deficiency alone, may drive muscle pathology in CAC. Such complexity challenges the conventional view that vitamin D supplementation is universally beneficial in cancer patients, particularly those with active muscle wasting [11].

Genetic polymorphisms in *VDR* may further modify individual responses to vitamin D signalling. The rs731236 polymorphism has been associated with altered receptor transcriptional activity or stability, although its functional consequences appear to be context-dependent. Tomei et al. (2020) [56] conducted an interventional study in a healthy cohort composed mainly of women of Arab ancestry to assess the association between response to vitamin D supplementation and genetic variants in vitamin D-related genes. They found that the rs731236 GG genotype was significantly associated with a “vitamin D sufficiency” state [serum 25(OH)D levels ≥30 ng/mL], and those with this genotype had a higher response to vitamin D supplementation.

Several studies have explored associations between rs731236 and musculoskeletal traits. The polymorphism has been associated with hand grip strength in schoolchildren [57]. However, other investigations did not detect significant associations with muscle strength or sarcopenic traits, suggesting that other *VDR* variants, such as rs1544410 (BsmI) and rs7975232 (ApaI), may contribute more strongly to these phenotypes [16,58,59,60]. Notably, rs731236 frequently occurs in linkage disequilibrium with these polymorphisms, indicating that haplotypic effects may amplify its functional consequences [16]. Among Chinese Han adults, the rs731236 variant was associated with differential adaptations to resistance training, with sex-specific effects. Namely, in women, AA genotype carriers achieved greater strength and power gains, whereas AG genotype carriers demonstrated more pronounced improvements in body composition [61]. Ovesjö et al. (2016) reported that carriers of the CC genotype (or GG genotype, depending on the DNA strand) had a fourfold increased risk of developing myopathy compared to individuals with the TT genotype (or AA genotype) [62]. A follow-up study by the same research group using in vitro experiments on primary human myoblasts suggested that the polymorphism does not directly influence the inhibitory effects of vitamin D on myoblast proliferation or differentiation. However, it has been shown that the GG genotype correlates with a higher number of VDREs, which can lead to an exaggeration of the vitamin D response, suppressing genes related to myogenic fusion, thus preventing the repair of damaged muscle fibres [63]. In the present study, the GG genotype (previously associated with vitamin D sufficiency) was linked to a protective effect against CAC development. This finding appears to contrast the cumulative evidence in musculoskeletal diseases but can be reconciled through the “molecular paradox” of vitamin D signalling in the context of CAC, whereby inflammatory and metabolic dysregulation reprograms VDR activity, altering its effects on muscle homeostasis [11].

In addition to CAC susceptibility, *VDR* rs731236 was also associated with cancer patient survival in the overall cohort (regardless of CAC status), independent of clinical covariates such as patient sex, age, body mass index (BMI), and inflammatory indices [prognostic nutritional index (PNI) and neutrophil-to-lymphocyte ratio (NLR)] at the time of patient recruitment (i.e., CAC diagnosis assessment). This observation suggests that the polymorphism may influence prognosis through mechanisms extending beyond muscle wasting alone. Indeed, VDR signalling exerts pleiotropic systemic effects, interacting with multiple metabolic and immune pathways, including the regulation of inflammatory cytokine profiles, oxidative stress responses, mitochondrial function, insulin sensitivity and autophagy, all of which have been independently linked to cancer progression, treatment tolerance and survival [10]. Consequently, genetic variation in *VDR* may affect clinical outcomes through broad host-level mechanisms, irrespective of cachexia status. By contrast, no significant associations were observed in analyses stratified by CAC status, most likely reflecting the small subgroup sizes and limited statistical power rather than a true absence of effect. Hence, these findings should also be interpreted within the broader context of vitamin D biology.

Vitamin D deficiency is highly prevalent across diverse geographic regions, particularly among older individuals, and has been associated with reduced physical performance and increased mortality risk [13,64]. This deficiency is even more common in patients with advanced cancer and correlates strongly with greater pain burden, fatigue, and increased opioid requirements [65,66,67]. Although most investigations of rs731236 have focused on cancer susceptibility, accumulating evidence suggests that this polymorphism may also influence prognosis, albeit with inconsistent findings across tumour types. In a breast cancer cohort, carriers of the GG genotype exhibited significantly higher cancer-specific mortality [36]. Similarly, in non-resectable lung cancer, rs731236 was an independent predictor of progression-free survival and OS, with GG carriers again showing poorer outcomes [68]. Conversely, a study of metastatic colorectal cancer reported longer OS among patients with the GG genotype compared with A allele carriers in a discovery cohort. Nevertheless, this association was not confirmed in an independent validation cohort [69]. In line with this heterogeneity, a large pooled colorectal cancer analysis reported no significant association between rs731236 and either cancer-specific or all-cause mortality [70], and a study in head and neck squamous cell carcinoma likewise found no statistically significant survival effects [71]. These heterogeneous results, including those from the present study, suggest that the prognostic impact of rs731236 is tumour- and context-dependent, likely influenced by factors such as inflammatory burden, tumour microenvironment, patient comorbidities, and CAC prevalence. Notably, in this study cohort, which encompassed patients with diverse tumour types, stratified analyses were not feasible due to small subgroup sizes, limiting statistical power and the ability to detect tumour-specific effects.

Overall, this study provides a personalised medicine perspective, emphasising the importance of host genetic factors when evaluating supportive or nutritional interventions in patients with cancer. Despite the promising findings, this study had some limitations that should be noted. The single-centre design and modest sample size may have limited statistical power and generalisability. Additionally, the relatively small cohort precluded subgroup analyses according to tumour type, stage and treatment modality, which are particularly relevant given the multifactorial nature of CAC. Furthermore, due to the retrospective design, circulating vitamin D levels (i.e., vitamin D status) were not available, which prevented direct assessment of genotype–phenotype relationships and their inclusion in the multivariable Cox regression analysis. Nevertheless, the study had important strengths, including the application of two cachexia classification systems and robust genotyping methodology, which enhance the reliability of the observations. Future research incorporating larger, multicentre cohorts, biochemical profiling of vitamin D status, and comprehensive assessment of cachexia-related parameters, such as skeletal muscle mass, longitudinal weight loss trajectories, C-reactive protein, and serum albumin, alongside evaluation of muscle proteolytic markers, functional assessments and mechanistic analyses of the *VDR* rs731236 polymorphism, is warranted. Such studies will be critical to clarify the mechanistic and clinical implications of *VDR* variants and to explore the potential for genotype-guided therapeutic strategies. Additionally, investigating other *VDR* polymorphisms may further expand understanding of how inherited variation influences muscle homeostasis and cancer outcomes. Finally, future studies should address potential sex-specific effects of rs731236 on CAC development and cancer survival, which could not be assessed in the present study due to an unbalanced sex distribution and limited statistical power in subgroup analyses.

## 4. Materials and Methods

### 4.1. Study Design and Participants

A retrospective cohort of cancer patients treated at the Portuguese Oncology Institute of Porto (IPO Porto, Portugal) was assembled, comprising individuals initiating or receiving first-line treatment, as well as those under palliative care. The cohort included participants of European descent aged 19 years or older, with an Eastern Cooperative Oncology Group Performance Status (ECOG-PS) of ≤3. Exclusion criteria comprised patients who requested a second medical opinion, were receiving medication for anorexia, had cognitive deficits, faced language barriers or refused to participate in the study. Based on these criteria, a total of 140 cancer patients were consecutively recruited between March 2023 and May 2024, with a mean follow-up of 76.3 ± 4.0 weeks. All participants provided informed consent.

At study enrolment, cachexia-related data were obtained by an experienced dietitian (A.C.L.S.) and CAC status was determined according to the Fearon criteria and CASC-IN tool, incorporating information on involuntary weight loss during the preceding six months, BMI, anorexia, and markers of systemic inflammation [72]. Anthropometric measurements were also taken, including weight on a digital scale (iHealth Nexus HS2S^®^, iHealth Labs^®^, Sunnyvale, CA, USA) and height on a stadiometer. To evaluate the participants’ nutritional health and inflammatory profiles, the study employed NLR and the PNI. The PNI is quantified by adding the serum albumin level (g/L) to five times the absolute lymphocyte count [serum albumin value (g/L) + 5X total lymphocytes (10^9^/L)]. For NLR, it is calculated as the absolute neutrophil count divided by the absolute lymphocyte count [73]. These ratios are relevant in the study of CAC, as systemic inflammation is the fundamental mechanism driving that condition [74].

**Figure 4:**
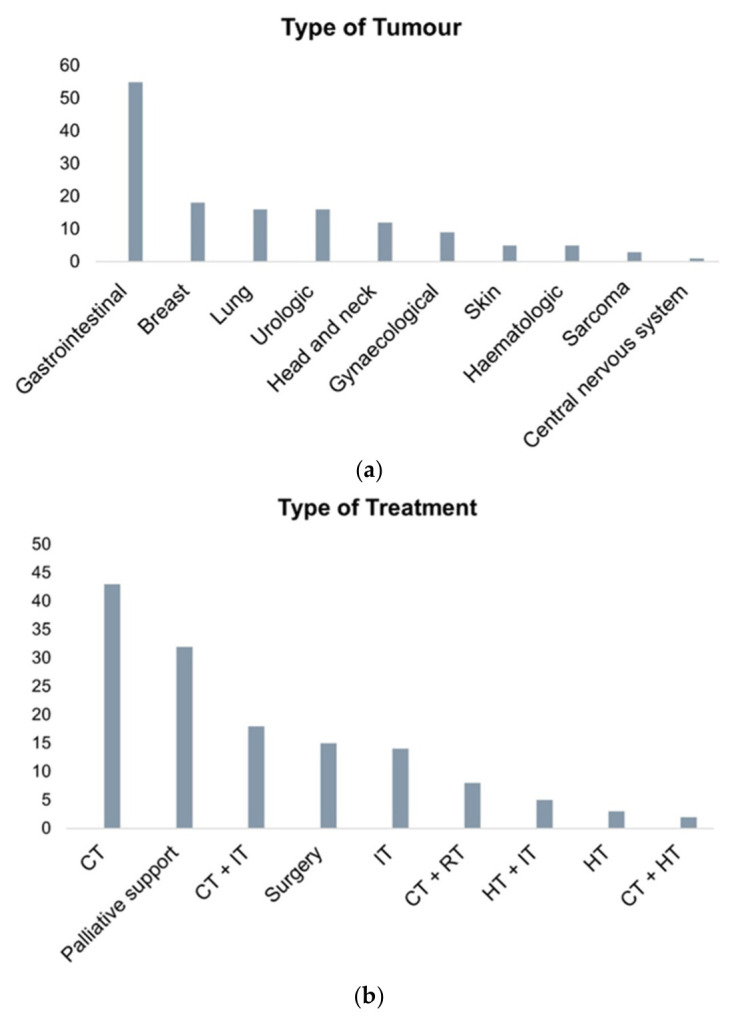
Distribution of malignant disease types (**a**) and cancer treatments (**b**) in the Study Cohort. Abbreviations: CT, chemotherapy; HT, hormonotherapy; IT, immunotherapy; RT, radiotherapy.

On the same day of patient recruitment and CAC diagnosis assessment, blood samples were also collected, and clinical history and demographic information were retrieved from patients’ medical records. Women comprised 54.3% (N = 76) of the study population. At enrolment, the mean age was 63.08 ± 1.1 years, with 47.1% (N = 66) of patients being over 63 years old. The mean BMI was 26.02 ± 0.44. A BMI < 26 kg/m^2^ was observed in 52.1% (N = 73) of participants. Concerning performance status, 48.3% (N = 57) of patients had an ECOG-PS of 0, 33.1% (N = 39) had a score of 1, 16.1% (N = 19) had a score of 2, and 2.5% (N = 3) had a score of 3. Metastatic disease was present in 64.3% (N = 90) of the cohort, while 5.7% (N = 8) reported a history of other malignancies. Details regarding tumour types and treatment modalities are presented in Figure 4.

This study was approved by the Human Research Ethics Committee of IPO Porto (CES. 131/022, approved on 28 July 2022).

### 4.2. Sample Processing and Genomic DNA Extraction

At enrolment, peripheral blood was obtained from participants through standard venous blood collection. The specimens were placed in EDTA-anticoagulated tubes to prevent coagulation.

Extraction of genomic DNA was performed using the MagaBio Plus Virus DNA/RNA Purification Kit II (BSC71S1E, Bioflux^®^, Tokyo, Japan) in combination with the MGISP-NE32 automated nucleic acid extraction system (MGI Tech^®^ Guangdong, China), strictly adhering to the supplier’s protocol. The yield and purity of the isolated DNA were evaluated spectrophotometrically using a NanoDrop Lite instrument (Thermo Fisher Scientific, Waltham, MA, USA). Verified DNA samples were subsequently stored at −20 °C until further analysis.

### 4.3. SNP Genotyping

Genotypic analysis of the *VDR* rs731236 polymorphism was performed using a StepOnePlus real-time PCR platform (Applied Biosystems, Carlsbad, CA, USA) with a fluorescence-based TaqMan allelic discrimination assay. The preparation of the amplification reactions was previously described [75,76,77,78,79]. Briefly, the total volume for each reaction was 6 μL, comprising 2.5 μL of TaqPath™ ProAmp™ Master Mix at working concentration (Applied Biosystems^®^, Foster City, CA, USA), 0.125 μL of the specific TaqMan^®^ SNP Genotyping Assay [C___2404008_10 targeting the *VDR* rs731236 variant; Supplementary Table S1; (Applied Biosystems^®^, Foster City, CA, USA], 1 μL of template DNA, and nuclease-free water to reach the final reaction volume. Thermal cycling parameters followed a previously established protocol [77].

To monitor potential contamination, two no-template controls (i.e., negative controls) were incorporated into each run. In addition, genotyping reproducibility was validated by repeating the analysis on 20% of randomly selected samples. Interpretation of genotype calls was performed independently by three investigators who were unaware of the corresponding clinical and pathological information.

### 4.4. Statistical Analysis

Quantitative analyses and figure generation were carried out using IBM SPSS Statistics for Windows, version 30.0 (IBM Corp., Armonk, NY, USA) and Microsoft Excel (Microsoft Corp., Redmond, WA, USA), respectively.

Genotype frequencies observed in the study cohort were contrasted with reference data from the Iberian population available through the Ensembl database (https://www.ensembl.org/index.html, last accessed on 28 December 2025). Conformity between observed and expected genotype distributions was examined by testing for HWE using the χ^2^ test of independence.

The distributional properties of continuous variables were assessed using the Kolmogorov–Smirnov test. Depending on whether the variables followed a parametric or non-parametric distribution, data were categorised based on either mean or median values, respectively. Regarding PNI and NLR, initially, patients were ranked according to each marker and divided into tertiles. These three-tier classifications were subsequently combined into binary categories. For PNI, the lowest tertile (≤44.2) was designated as the low group, whereas the remaining two tertiles constituted the high group. In contrast, NLR values were considered low when falling within the first two tertiles (<3.6) and high when belonging to the upper tertile (≥3.6).

Cachexia status was assessed using the Fearon criteria, the CASC-IN classification system, and a combined approach. In the latter, patients were assigned to a group only if the Fearon and CASC-IN systems yielded the same classification. For these three diagnostic strategies, cachexia status was evaluated in two ways: firstly, using three categories (non-cachectic, pre-cachectic and cachectic); and secondly, via a binary classification where pre-cachectic and cachectic patients were grouped as cachexia-positive.

Relationships between the *VDR* rs731236 variant and CAC occurrence, as well as demographic and clinicopathological parameters, were evaluated using the χ^2^ test or Fisher’s exact test, as appropriate based on expected cell frequencies. Logistic regression analyses were conducted to estimate the CAC risk (OR and 95% CIs) associated with each SNP genotype.

The clinical outcome evaluated in this study was OS, which was defined as the time elapsed from study inclusion (corresponding to cachexia assessment) to death from any cause or to the most recent follow-up for censored cases. Survival outcomes related to *VDR* genetic variation were explored using Kaplan–Meier methodology. Group comparisons were performed with either the log-rank test or the Tarone–Ware test, selected according to compliance with proportional hazard assumptions and the genetic inheritance model best supported by preliminary survival curve inspection. The impact of the SNP on the risk of patient death was also examined via Cox regression analysis. Multivariable Cox analysis was conducted, adjusting for relevant demographic and clinicopathological parameters. Due to cohort size, subgroup analyses according to tumour type and cancer treatment approaches were not conducted. To evaluate data stability and reliability, bootstrap resampling (1000 iterations) was carried out.

All hypothesis testing was conducted using two-tailed procedures, and statistical significance was established at a threshold of *p* < 0.05.

## 5. Conclusions

CAC represents one of the most severe and clinically challenging complications in cancer patients and it is therefore important to identify biological determinants that influence both its susceptibility and prognosis. Hence, this real-world study examined the implications of *VDR* rs731236 in a cohort of cancer patients. Although these findings should be interpreted cautiously in light of the study’s limitations, they provide further evidence that this polymorphism is significantly associated with both CAC risk and patient survival. While routine genetic screening is not yet standard practice in oncology supportive care, these findings add to the growing body of evidence emphasising the importance of host genetic factors in shaping clinical outcomes. Future studies integrating *VDR* rs731236 with assessments of circulating vitamin D status and targeted supportive interventions are warranted to clarify the translational relevance of these associations. Moreover, further research is needed to determine the clinical effectiveness of vitamin D supplementation in preventing or mitigating active CAC. Importantly, the broader literature suggests that the biological and clinical effects of *VDR* rs731236 and vitamin D signalling are context-dependent. While vitamin D is widely recognised for its pro-regenerative actions in skeletal muscle under physiological conditions, these benefits may not translate directly to pathological states such as CAC. Likewise, the prognostic significance of rs731236 remains inconsistent across malignancies. Overall, these findings seem to reinforce the concept that vitamin D signalling in cancer is highly context-dependent and that genetic variation in *VDR* may represent a clinically relevant modifier of cancer outcomes. Pending external validation in larger cohorts, *VDR* rs731236 may serve as a useful biomarker for CAC risk stratification and prognostication, particularly within the evolving field of liquid biopsy.

## Figures and Tables

**Table 1:** Distribution of *VDR* rs731236 genotypes across groups (no cachexia, pre-cachexia, and cachexia) as defined by FEARON criteria, CASC-IN, and combined assessment tools.

Diagnostic Approach		*VDR* rs731236 Genotypes						
		Codominant			Recessive		Dominant	
		GG N (%)	AG N (%)	AA N (%)	GG N (%)	AG + AA N (%)	GG + AG N (%)	AA N (%)
**Fearon** **criteria**	No CAC	22 (81.5)	34 (58.6)	27 (49.1)	22 (81.5)	61 (54.0)	56 (65.9)	27 (49.1)
	Pre-CAC	3 (11.1)	7 (12.1)	5 (9.1)	3 (11.1)	12 (10.6)	10 (11.8)	5 (9.1)
	CAC	2 (7.4)	17 (29.3)	23 (41.8)	2 (7.4)	40 (35.4)	19 (22.4)	23 (41.8)
	*p*-Value	**0.030**			**0.012**		**0.048**	
**CASC-IN**	No CAC	22 (81.5)	37 (63.8)	27 (50.9)	22 (81.5)	64 (57.7)	59 (69.4)	27 (50.9)
	Pre-CAC	3 (11.1)	4 (6.9)	5 (9.4)	3 (11.1)	9 (8.1)	7 (8.2)	5 (9.4)
	CAC	2 (7.4)	17 (29.3)	21 (39.6)	2 (7.4)	38 (34.2)	19 (22.4)	21 (39.6)
	*p*-Value	**0.027**			**0.028**		0.075	
**Combined tools**	No CAC	20 (87.0)	33 (62.3)	24 (51.1)	20 (87.0)	57 (57.0)	53 (69.7)	24 (51.1)
	Pre-CAC	1 (4.3)	3 (5.7)	2 (4.3)	1 (4.3)	5 (5.0)	4 (5.3)	2 (4.3)
	CAC	2 (8.7)	17 (32.1)	21 (44.7)	2 (8.7)	38 (38.0)	19 (25.0)	21 (44.7)
	*p*-Value	**0.026**			**0.011**		0.081	

Bold values were deemed statistically significant. Abbreviations: CAC, cancer-associated cachexia; N, number of patients.

**Table 2:** Distribution of *VDR* rs731236 genotypes by disease status (no cachexia vs. pre-cachexia/cachexia) according to FEARON criteria, CASC-IN, and combined assessment tools.

Diagnostic Approach		*VDR* rs731236 Genotypes						
		Codominant			Recessive		Dominant	
		GG N (%)	AG N (%)	AA N (%)	GG N (%)	AG + AA N (%)	GG + AG N (%)	AA N (%)
**Fearon** **criteria**	No CAC	22 (81.5)	34 (58.6)	27 (49.1)	22 (81.5)	61 (54.0)	56 (65.9)	27 (49.1)
	CAC + Pre-CAC	5 (18.5)	24 (41.4)	28 (50.9)	5 (18.5)	52 (46.0)	29 (34.1)	28 (50.9)
	*p*-Value	**0.019**			**0.017**		0.072	
**CASC-IN**	No CAC	22 (81.5)	37 (63.8)	29 (52.7)	22 (81.5)	66 (58.4)	59 (69.4)	29 (52.7)
	CAC + Pre-CAC	5 (18.5)	21 (36.2)	26 (47.3)	5 (18.5)	47 (41.6)	26 (30.6)	26 (47.3)
	*p*-Value	**0.040**			**0.045**		0.069	
**Combined tools**	No CAC	20 (87.0)	33 (62.3)	24 (51.1)	20 (87.0)	57 (57.0)	53 (69.7)	24 (51.1)
	CAC + Pre-CAC	3 (13.0)	20 (37.7)	23 (48.9)	3 (13.0)	43 (43.0)	23 (30.3)	23 (48.9)
	*p*-Value	**0.014**			**0.015**		0.059	

Bold values were deemed statistically significant. Abbreviations: CAC, cancer-associated cachexia; N, number of patients.

**Table 3:** Multivariable Cox analysis on the risk of death.

Variable	aHR	95%CI	*p*-Value	Bootstrap *p*-Value
*VDR* rs731236 (GG vs. AA/AG *)	0.23	**0.07–0.75**	**0.015**	**0.006**
Patient sex (female vs. male *)	1.00	0.53–1.90	0.992	0.990
Patient age at recruitment (≥63 vs. <63 years *)	1.67	0.88–3.115	0.120	0.141
BMI at recruitment (<26 vs. ≥26 kg/m^2^ *)	1.41	0.76–2.61	0.273	0.276
PNI (≤44.2 vs. >44.2 *)	2.01	**1.03–3.92**	**0.040**	0.058
NLR (≥3.6 vs. <3.6 *)	3.56	**1.79–7.06**	**<0.001**	**0.002**

Bold values were deemed statistically significant. *—reference group. Abbreviations: aHR, adjusted hazard ratio; BMI, body mass index; CI, confidence interval; NLR, neutrophil-to-lymphocyte ratio; PNI, prognostic nutritional index.

## Data Availability

The data presented in this study are available on request from the corresponding author due to patient data protection.

## Supplementary Materials

The following supporting information can be downloaded at: https://www.mdpi.com/article/10.3390/ijms27135816/s1.

## Abbreviations

The following abbreviations are used in this manuscript:

aHR	Adjusted hazard ratio
BMI	Body mass index
CAC	Cancer-associated cachexia
CI	Confidence interval
ECOG-PS	Eastern Cooperative Oncology Group Performance Status
HR	Hazard ratio
HWE	Hardy–Weinberg equilibrium
IGF-1	Insulin-like growth factor 1
IL-6	Interleukin-6
mRNA	Messenger ribonucleic acid
NLR	Neutrophil-to-lymphocyte ratio
OR	Odds ratio
OS	Overall survival
PNI	Prognostic nutritional index
SNP	Single-nucleotide polymorphism
TNF-α	Tumour necrosis factor-α
VDR	Vitamin D receptor
VDREs	Vitamin D response elements
