# Supplementary material for: Vitamin D Receptor rs731236 Polymorphism Modulates Cancer Cachexia Susceptibility and Overall Survival: A Real-World Study on Context-Dependent Vitamin D Signalling
Source: Int J Mol Sci. 2026 Jun 27;27(13):5816. doi: 10.3390/ijms27135816 (PMC13361905; doi:10.3390/ijms27135816)
Supplement: Supplementary file 1 [file ijms-27-05816-s001.zip › ijms-4398486-supplementary.pdf]

Supplementary Material

Supplementary Table S1: Description of the TaqMan assay used in the study

**SNP**	**TaqMan Assay ID**	**Allele [VIC/FAM]**	**Context Sequence [VIC/FAM]**	**Location**
VDR rs731236	C___2404008_10	A/G	TGGACAGGCGGTCCTGGATGGCCTC**[A/G]**ATCAGCGCGGCGTCCTGCACCCCAG	Chr.12:47844974
